# Clinical Impact of Digitalis Therapy in a Large Multicenter Cohort of CRT-Recipients

**DOI:** 10.3390/jcdd11060173

**Published:** 2024-06-03

**Authors:** Julia W. Erath, Nikolett Vigh, Balazs Muk, Carsten W. Israel, Sarah Keck, David Pilecky, Gabor Z. Duray, Mate Vamos

**Affiliations:** 1 Department of Cardiology, Goethe University Hospital, 60590 Frankfurt am Main, Germany; sarah-keck@gmx.de (S.K.);; 2 Department of Cardiology, Medical Centre, Hungarian Defense Forces, 1062 Budapest, Hungary; vighnik@gmail.com (N.V.); balaszmukmd@gmail.com (B.M.); gabor.duray@yahoo.com (G.Z.D.); 3 Department of Adult Cardiology, Gottsegen National Cardiovascular Center, 1096 Budapest, Hungary; pileckyd@gmail.com; 4 Department of Cardiology, Evangelical Hospital, 33617 Bielefeld, Germany; carsten.israel@bethel.de; 5 Cardiac Electrophysiology Division, Cardiology Center, Department of Internal Medicine, University of Szeged, 6725 Szeged, Hungary

**Keywords:** digitalis, digoxin, digitoxin, cardiac resynchronization therapy, CRT-D, defibrillator, congestive heart failure, mortality, CRT-response

## Abstract

(1) Introduction: Digitalis use in patients with severe heart failure is controversial. We assessed the effects of digitalis therapy on mortality in a large, observational study in recipients of cardiac resynchronization therapy (CRT). (2) Methods: Consecutive patients receiving a CRT-defibrillator in three European tertiary referral centers were enrolled and followed-up for a mean 37 months ± 28 months. Digitalis use was assessed at the time of CRT implantation. A multivariate Cox-regression model and propensity score matching were used to determine all-cause mortality as the primary endpoint. CRT-response (defined as improvement of ≥1 NYHA class), echocardiographic improvement (defined as improvement of LVEF of ≥ 5%) and incidence of ICD shocks and rehospitalization were assessed as secondary endpoints in a subgroup of patients. (3) Results: The study comprised 552 CRT-recipients with standard indications, including 219 patients (40%) treated with digitalis. Compared to patients without digitalis, they had more often atrial fibrillation, poorer LVEF and a higher NYHA class (all *p* ≤ 0.002). Crude analysis of all-cause mortality demonstrated a similar relative risk of death for patients with and without digitalis (HR = 1.14; 95% CI 0.88–1.5; *p* = 0.40). After adjustment for independent predictors of mortality, digitalis therapy did not alter the risk for death (adjusted HR = 1.04; 95% CI 0.75–1.45; *p* = 0.82). Furthermore, in comparison to 286 propensity-score-matched patients, mortality was not affected by digitalis intake (propensity-adjusted HR = 1.11; 95% CI 0.72–1.70; *p* = 0.64). A CRT-response was predominant in digitalis non-users, concerning both improvement of HF symptoms and LVEF (NYHA *p* < 0.01; LVEF *p* < 0.01), while patients on digitalis had more often ventricular tachyarrhythmias requiring ICD shock (*p* = 0.01); although, rehospitalization for cardiac reasons was significantly lower among digitalis users compared to digitalis non-users (HR = 0.58; 95% C. I. 0.40–0.85; *p* = 0.01). (4) Conclusions: Digitalis therapy had no effect on mortality, but was associated with a reduced response to CRT and increased susceptibility to ventricular arrhythmias requiring ICD shock treatment. Although, digitalis administration positively altered the likelihood for cardiac rehospitalization during follow-up.

## 1. Introduction

Hospital admission rates for congestive heart failure were the highest in 2015 in Germany, with a rate of 3.7 per 1000 inhabitants compared to the European average rate of 2.4 per 1000 inhabitants, despite high-quality primary care causing tremendous costs for the health care system [1]. One solution might be cardiac resynchronization therapy (CRT) for a selected group of heart failure patients, which was proved to reduce both hospitalization rates [2,3,4,5,6,7] and mortality [8] in multiple trials. Therefore, current guidelines recommend CRT implantation for heart failure (HF) patients with reduced LVEF as a class IA indication in case of a left bundle branch block (LBBB) of ≥150 ms and in case of a complete heart block in AF or sinus rhythm to avoid right ventricular pacing as well as a class IIA indication for shorter QRS width (130–149 ms) in sinus rhythm [9,10]. Digitalis is currently recommended as a class IB [11] indication to establish rate control in patients with atrial fibrillation and HF with an LVEF ≤ 40% and as second-line treatment in patients with suboptimal heart rate control or worsening symptoms under a beta-blocker and/or non-dihydropyridine calcium-antagonist treatment [11]. To treat HF, the ESC HF guidelines recommend digoxin as a class II B indication to treat symptomatic patients with reduced LVEF in sinus rhythm, despite guideline-directed medical therapy, to reduce the risk of hospitalization (both all-cause and HF hospitalizations) [10]. Only the latter indication has been investigated in the randomized controlled DIG trial. This trial, published in the nineties, failed to show a reduction in mortality, but showed lower hospitalization rates in patients randomized to digitalis therapy instead [12]. Within the last decade, medical heart failure therapy has been broadened to the widespread use of angiotensin-converting-enzyme inhibitors (ACEI), aldosterone antagonists, angiotensin-neprilysin-inhibitors (ARNI) and, lately, SGLT-2 inhibitors, which all proved to reduce both mortality and heart-failure-related events [13,14,15]. In two previous meta-analyses comprising over 1,000,000 patients, we could demonstrate that digoxin is associated with increased mortality in both patients with AF and/or HF [16,17]. When aiming for targeted therapeutic serum digoxin levels by regular blood controls, digoxin use might not lead to higher mortality rates compared to digoxin non-users in an HF patient collective [18]. It is also noteworthy that even with an extremely close monitoring strategy, it is only possible to maintain serum digoxin levels in the target range in less than 50% of patients [18]. ICD patients also showed a higher mortality rate when allocated to digitalis therapy compared to patients not on digitalis [19]. Furthermore, digitalis therapy might induce fatal ventricular arrhythmias, as CRT and ICD patients experienced more high-rate VT/VF episodes in a subgroup analysis of the MADIT-CRT trial [20].

Whether digitalis therapy has an impact on mortality and CRT-response in this context was investigated in this large, non-randomized multicenter CRT-D study.

## 2. Methods

**Patient population:** This non-randomized tricenter observational study is based on the analysis of data collected from consecutive patients who received a CRT-defibrillator at the J. W. Goethe University (Frankfurt, Germany), at the Medical Centre of the Hungarian Defense Forces (Budapest, Hungary) and at the Bethel-Clinic (Bielefeld, Germany) between 2005 and 2019. All analyzed patients were followed-up at the institution where the implantation was performed. Devices from various manufacturers were used (Medtronic, Minneapolis, MN, USA; St. Jude Medical/Ventritex, St. Paul, MN, USA; Guidant/Boston Scientific, St. Paul, MN, USA; ELA/Sorin, Milano, Italy; Biotronik, Berlin, Germany). The study was approved by the IRB of the J.W. Goethe University and it conforms to the ethical guidelines of the Declaration of Helsinki.

**Data collection:** Data were retrospectively collected from the index hospitalization at the time of initial CRT-D implantation and at each follow-up visit, which took place every six months or at the time of unscheduled visits in the out- or in-patient clinic. Data collection included patient characteristics such as age, indication for CRT, LVEF and LVEDD and relevant comorbid conditions. LVEF was evaluated by experienced cardiologists independently (not aware of the purpose of the study), as well as by automatic measurements (GE Healthcare, AutoEF, Chicago, IL, USA). ECG parameters, such as atrioventricular (AV) conduction or QRS width before and after CRT-implantation, as well as biventricular pacing properties, were additionally assessed. NYHA classification was calculated at implantation and every follow-up visit. Pertinent medication use (beta-blockers, ACE-inhibitors or angiotensin receptor blockers, diuretics, aldosterone antagonists, digitalis glycosides, antiarrhythmic drugs) was documented. Digitalis was used to treat heart failure and/or to control heart rate in atrial fibrillation, according to current guideline recommendations [10,11]. Data were also collected from blood tests. Serum digitalis levels were checked at the discretion of the treating physician. All relevant information was entered into a customized database. For missing data, particularly in case of missed follow-up visits, family members, treating physicians or other hospitals were contacted to retrieve the missing information.

**Outcomes:** The primary outcome measure was time to all-cause mortality. Cause-specific mortality was defined according to the Hinkle and Thaler classification [21]. Secondary endpoints were CRT-response, defined as an improvement of ≥1 NYHA class and improvement of LVEF by at least 5% within the first year after CRT-D implantation. Moreover, QRS shortening by at least 10 ms during follow-up was assessed. The incidences of appropriate and inappropriate ICD shocks, as well as rehospitalization, were also collected and compared between the two groups.

**Statistics:** Statistical analysis was performed using the SPSS version 22 program (IBM, Chicago, IL, USA) and R core (GNU GPL, Tulsa, OK, USA). Baseline characteristics were compared using the Wilcoxon Mann–Whitney U-test (continuous variables) and the Chi^2^-test or the Fisher exact test (categorical variables). Survival analysis was performed using a Kaplan–Meier analysis. Survival curves were compared using the log-rank test and the Wald test for the Cox proportional hazard model. Crude and adjusted hazard ratios (HR) with 95% confidence intervals for digitalis use were calculated for potential confounding factors including age, gender, primary/secondary prevention indication, ischemic/non-ischemic heart disease, NYHA class, LVEF, upgrade/de novo implantation, hypertension, QRS width, documented atrial fibrillation, diabetes mellitus, chronic renal disease and amiodarone use. Additionally, propensity score matching was performed using the nearest neighborhood method with a 1:1 matching ratio and a caliper of 0.2. Within this matched cohort, a Kaplan–Meier survival analysis was repeated, and groups were compared using a log-rank test. Only two-sided tests were used and *p*-values of *p* < 0.05 were considered statistically significant.

## 3. Results

**Patient population:** A total of 575 heart failure patients received a CRT-D device for primary or secondary prevention from sudden cardiac death in 2005–2015 in Frankfurt, Bielefeld and Budapest. Of those, 552 patients were regularly followed-up for a mean 37 ± 28 months in the in- and out-patient clinics; these patients form the basis of this report.

Our patient collective consists of mainly men (77%), with a mean age of 67 ± 11 years and symptomatic heart failure due either to ischemic (n = 298; 54%) or non-ischemic heart disease (n = 254; 46%). Left ventricular function was severely reduced with a mean LVEF of 25 ± 7% and broad QRS was present in all patients, indicating intraventricular conduction disturbances (QRS width mean = 160 ± 28 ms), with 76% having left bundle branch block, 10% having right bundle branch block and 14% having a non-specific intraventricular conduction delay or wide-paced QRS based on the definition by the European Society of Cardiology [22]. The most frequent comorbidities were atrial fibrillation (n = 200; 36%), hypertension (n = 381; 69%), diabetes mellitus (n = 194; 35%) or chronic kidney disease (n = 284; 51%) (Table 1). In all, 219 patients received digitalis in addition to heart failure medication (40%; Table 2) and 333 patients did not (60%). Patients on digitalis were younger (mean age 64 ± 11 vs. 67 ± 11 years; *p* < 0.001) and had better renal function (median eGFR 63 vs. 54 mL/min, *p* = 0.004), but suffered from more advanced heart failure with a lower LVEF (mean 23 ± 7 vs. 27 ± 7%, *p* < 0.001) and more frequent NYHA III symptoms (71 vs. 57%, *p* = 0.002) compared to patients not on digitalis. Additionally, significantly more patients on digitalis had episodes of atrial fibrillation documented before CRT-D implantation (47 vs. 29%; *p* < 0.001).

**Figure 1:**
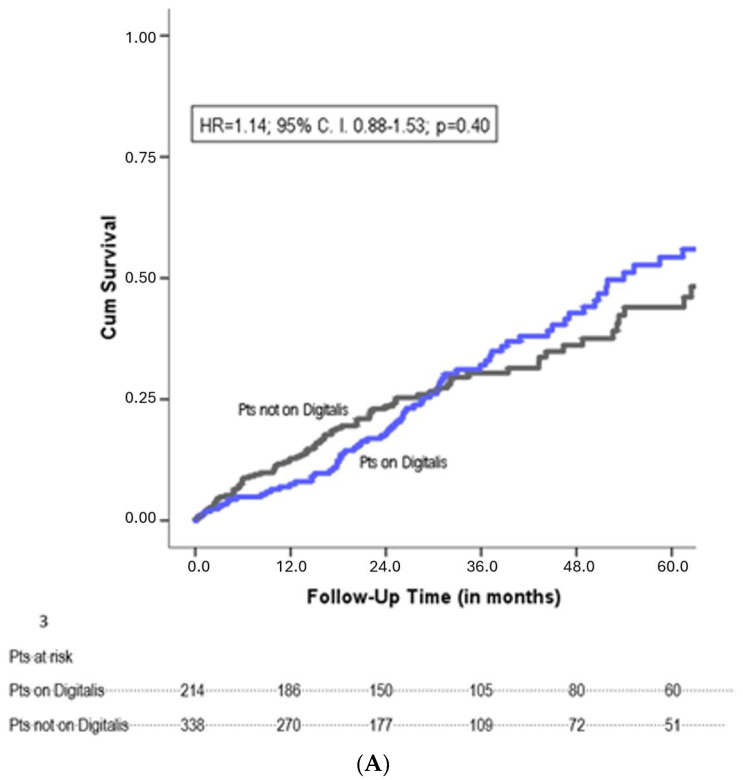

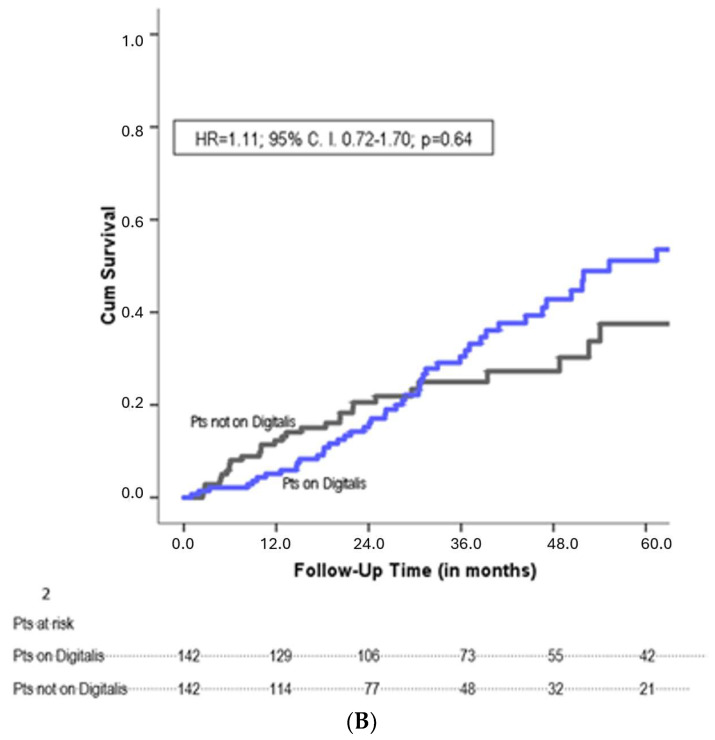
(**A**) Crude Kaplan–Meier analysis of mortality. (**B**) Adjusted Kaplan–Meier analysis of mortality showing the propensity-score-matched cohort.

**Figure 2:**
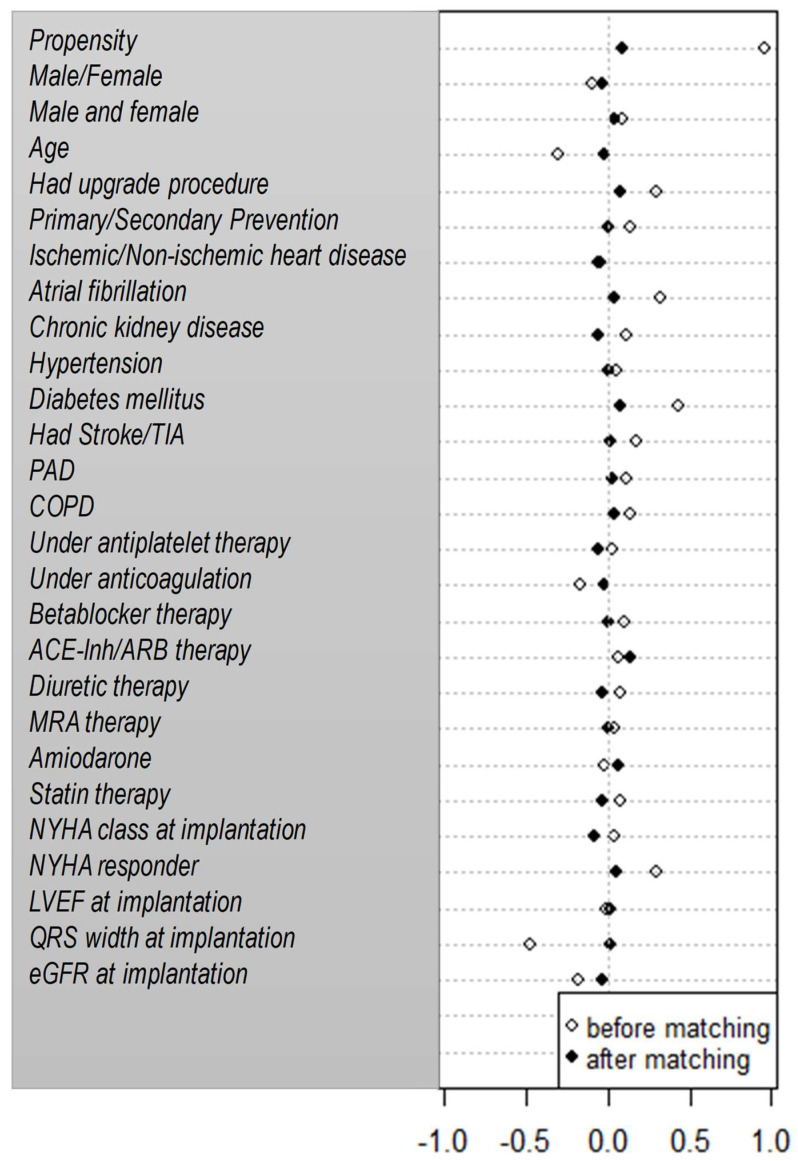
Dotplot of standardized mean differences for baseline characteristics between digoxin users and non-users, before and after propensity score matching via nearest neighborhood test.

**All-cause mortality:** During a follow-up period of 37 ± 28 months, 176 patients died (32%). A crude Kaplan–Meier analysis showed no significant difference in mortality between the two groups (HR = 1.14; 95% CI 0.88–1.53; *p* = 0.40) (Figure 1A). To correct for potential confounders, we performed a multivariate Cox-regression analysis for all baseline variables. This yielded the following independent predictors of mortality: age, male gender, chronic kidney disease, higher NYHA class and a CRT implanted as an upgrade of a pre-implanted pacemaker or defibrillator (Table 3). After adjustment, the risk for mortality continued to be similar among patients on digitalis and patients not on digitalis (adjusted HR = 1.04; 95% CI 0.75–1.45; *p* = 0.82). The propensity-score-matched cohort consisted of 286 patients, with 143 patients on digitalis and 143 patients not on digitalis, and had equally balanced baseline characteristics (Figure 2). Kaplan–Meier mortality analysis was performed and demonstrated no significant mortality difference between the two groups (propensity-adjusted HR = 1.11; 95% CI 0.72–1.70; *p* = 0.64) (Figure 1B).

**Cause-specific mortality:** In 23 of 114 death events, cause-specific mortality could not be assessed, as the circumstances surrounding death remained unknown (21%). Cardiac non-arrhythmic death due to progressive heart failure was the predominant cause of death in both patients receiving digitalis and patients not receiving digitalis (both 34%; *p* = 0.57), followed by non-cardiovascular deaths (21 vs. 23%), cardiac arrhythmic deaths (19 vs. 18%) and other cardiovascular deaths (3 vs. 9%) (*p* = n.s. for all comparisons) (Table 4).

**Rehospitalization and digitalis therapy:** Data on rehospitalization for cardiac reasons were only available in the Frankfurt and Budapest cohort of the patients (n = 447/552 patients). Of these, 179 patients were rehospitalized during the follow-up period of mean 37 ± 28 months, yielding an incidence of 40%. When adjusted to the use of digitalis glycosides, patients on digitalis had a significantly lower relative risk for rehospitalization compared to patients without digitalis medication (HR = 0.58; 95% C. I. 0.40–0.85; *p* = 0.01).

**Arrhythmia burden and digitalis therapy:** We assessed episodes of ventricular arrhythmias in a subgroup analysis of the Frankfurt cohort. Tachycardia information was available in 58% of the patients (n = 212). Ventricular arrhythmia (non-sustained/sustained VT and VF) occurred in 72 patients (32%), with a trend towards a higher incidence in patients on digitalis (n = 50; 38% vs. n = 22; 27%; *p* = 0.07); however, patients on digitalis developed significantly more frequent VT/VF episodes requiring ICD shock therapy (23% vs. 10%; *p* = 0.01). The incidence of inappropriate shock therapy was low and not different between the patient groups (8% vs. 5%; *p* = 0.50).

**CRT-response and digitalis therapy:** Out of 552 patients, 336 (61%) were CRT-responders during the 37 ± 28 month follow-up period. Of these, 125 patients were on digitalis (37%) and 211 patients were not (63%). Digitalis non-users showed significantly improved HF symptoms, measured by improvement in NYHA class by at least one class (HR = 0.57; 95% CI 0.47–0.70; *p* < 0.01) six months after CRT implantation. Pairwise echocardiographic measurements (baseline and 6 months follow-up) were available in 358 patients for LVEF (65%). In this subgroup analysis, digitalis intake decreased CRT-response rates, measured by improvement in LVEF by at least 5% at 6 months and a relative risk reduction of 40% (HR = 0.60; 95% CI 0.45–0.78; *p* < 0.01). Furthermore, QRS shortening (≥10 ms) was more often present in digitalis non-users (HR = 0.62; 95% C. I. 0.46–0.83; *p* = 0.001). Accordingly, biventricular pacing properties were significantly lower in patients on digitalis (92.5% ± 15.1% versus 95.5% ± 10.8%; *p* = 0.02).

**Serum digitalis levels throughout the study:** From the total of 219 patients on any digitalis substance, 86 patients (39%) had at least one serum digitalis level value available, and 29 patients (13%) had five or more digitalis level values. The mean serum digoxin level (in 50 patients) was 0.77 ± 0.61 ng/mL (min–max 0.0–6.8). The mean digitoxin level (in 36 patients) was 20.97 ± 9.1 ng/mL (min–max <0.15–40.0). Notably, three patients from these changed from digoxin to digitoxin during follow-up.

## 4. Discussion

**Main findings:** In our multicenter observational study comprising a large cohort of over 500 CRT-D recipients, digitalis therapy had no impact on mortality, but it negatively altered the likelihood to respond to CRT and was associated with an increased risk of ventricular tachyarrhythmias requiring ICD shock.

**Impact of digitalis on mortality:** Digitalis glycosides are either used to improve rate control in patients with atrial fibrillation, to lessen symptoms of advanced stage heart failure or for both indications, according to current guideline recommendations [10,11]. These guideline recommendations are based on a single randomized controlled trial conducted over twenty years ago with patients who were only receiving beta-blockers for heart failure therapy. Under these circumstances, digitalis therapy was associated with a 23% risk reduction for heart failure hospitalization [12], and this finding could be reproduced in this patient cohort by analyzing the data of 81% of the patients. In the meantime, substances like ACE-inhibitors, aldosterone antagonists, ARNI and, most recently, SGLT-2 inhibitors have revolutionized medical heart failure therapy, as they have been demonstrated to be able to reduce both heart-failure-related events and mortality [13,14,15]. In the PARADIGM-HF trial, McMurray and colleagues demonstrated a 16% relative risk reduction in mortality in 8442 patients and a 21% risk reduction in heart-failure-related events (both *p* ≤ 0.001) due to sacrubitril/valsartan compared to enalapril intake. This patient population suffered from severe heart failure with a mean LVEF of 29%, similar to our collective. Most interestingly, 30% of the patients in each treatment arm were on digitalis medication as well [14]. Notably, ARNI or SGLT-2 inhibitors were still not prescribed in a regular fashion to the patients of the current study. For CRT, the COMPANION trial showed a 41% relative risk reduction for heart-failure-related hospitalizations [3], and a 36% relative risk reduction for death in patients implanted with a CRT-defibrillator [4]. It is of note that 78–79% of the patients received digitalis in the randomized groups [3,4]. Multiple observational studies demonstrated an increased risk of death in heart failure patients with or without atrial fibrillation due to digitalis administration [18,19,20,21,23,24,25]. Relative risk for death ranged from 14% to 72% [18,20,21]; patients with severely impaired left ventricular function did not benefit from digoxin intake in further studies [23,25].

In view of such study reports, it is somehow unexpected that digitalis therapy had no impact on mortality in the current multicenter observational study of CRT-D recipients [26]. Notably, a subgroup analysis from the MADIT-CRT trial demonstrated that digitalis therapy does not alter the risk for death (HR = 0.93; 95% C. I. 0.67–1.32; *p* = 0.71).

Another possible explanation for the observation that mortality was not increased by digitalis in our study with CRT patients might be based on the fact that proarrhythmic effects of digitalis were less life threatening because all patients were fitted with a defibrillator. This hypothesis is supported by the finding that VT/VF episodes requiring ICD shock therapy were more frequent in patients with digitalis.

A further explanation for the better-than-expected survival rate could be the better controlled serum digitalis concentration in this cohort. We could provide at least one serum digitalis level value in 39% of the included patients. The majority of these patients were treated with digoxin, in whom the mean digoxin concentration proved to be 0.77 ng/mL. Based on a post hoc analysis of the DIG trial [27] and some later observational studies [18], digitalis plasma concentrations under 0.9 ng/mL might be less harmful or even have a neutral effect on all-cause mortality.

**Cause-specific mortality and arrhythmia:** In our CRT-D population, progressive heart failure was the predominating cause of death in 34% of the patients, followed by non-cardiovascular and cardiac arrhythmic deaths (22% and 18%). Our results are in line with the findings of a subgroup analysis of the COMPANION trial, where 44% of the patients died due to pump failure [25]. Digitalis therapy was not associated with either one of these death modes classified according to Hinkle and Thaler [21]. It is of common knowledge that digitalis therapy increases the risk for cardiac arrhythmias and causes atrial arrhythmias, conduction disturbances and multiple forms of ventricular tachycardia [28]. Patients on digitalis experienced numerically more ventricular arrhythmias in our collective. The number of ventricular tachyarrhythmias leading to appropriate shock was significantly higher among patients on digitalis (*p* = 0.01). Digitalis therapy was associated with an increased risk for ventricular arrhythmias, especially with heart rates ≥ 200 beats per minute in the MADIT-CRT subgroup analysis (HR = 1.41; 95% CI 1.14–1.75; *p* = 0.002; HR = 1.65; 95% CI 1.27–2.15; *p* ≤ 0.001) [20]. This finding is also supported by a previous analysis of a large ICD collective which demonstrated a significant 30% higher risk of ICD shock therapy in patients on digitalis [19].

**CRT-response and digitalis therapy:** Approximately 60–70% of CRT-recipients respond to CRT-therapy by improvement of at least one NYHA class, improvement in a 6 min walk test or by increasing LVEF or decreasing LVEDD, respectively [2,3,4,5,6,8]. To the best of our knowledge, this is the first study that demonstrates that the CRT-response was negatively affected by digitalis therapy, concerning both improvement in NYHA functional class and improvement in LVEF. Consecutively, the biventricular pacing percentage was significantly lower in patients on digitalis. Although we observed normal digoxin levels in a subset of patients in our study, the reduced CRT-response might be explained, at least in part, by results of a preclinical study showing that toxic effects of digoxin can even be detected through subtherapeutic levels negatively affecting intracellular calcium homeostasis in the myocardium [29]. In contrast, a recently published retrospective study on 297 HF patients in sinus rhythm compared their cardiovascular endpoints (HF hospitalization, heart transplantation and all-cause mortality) based on receiving treatment with digitalis versus receiving no treatment with digitalis [26], showing no difference in CRT-responder rates. Furthermore, it is debatable whether the increased morbidity in our study population and higher patient number might have contributed to a significantly different response rate to CRT, based on digitalis treatment. It should also be noted that patients on digitalis had less often QRS narrowing in our analysis, which can also explain the decreased echocardiographic and clinical response in this patient group [30].

## 5. Limitations

Our data collection was retrospective; hence, all potential limitations of such a design apply to this analysis. We aimed to minimize potential confounding by carefully adjusting data to important patient characteristics found on univariate and multivariate analyses and we, therefore, performed a propensity-score-matched analysis. Despite this, residual confounding cannot be entirely excluded. Digitalis use was assessed at CRT-D implantation, but not during follow-up or at time of death. Serum digitalis levels were checked at the discretion of the treating physician, resulting in incomplete data. Furthermore, CRT-response was mainly assessed clinically by improvement of NYHA functional class, which is a rather subjective parameter. Echocardiographic data were not available for the whole study collective. Moreover, data on ICD shock discharge were only available for the Frankfurt CRT-cohort, and rehospitalization was only assessed in the Frankfurt and Budapest cohort. That is why the latter analysis/analyses of secondary endpoints, i.e., “CRT-response, ICD shock treatment and rehospitalization”, only reflect a subgroup of the patient cohort and were not adjusted to any potential confounders. Strengths of our study consist of the large patient cohort, the long follow-up duration and the heterogenous, real-world, multicenter collective.

## 6. Conclusions

Digitalis therapy had no effect on mortality, but was associated with a reduced response to CRT and increased susceptibility to ventricular arrhythmias requiring ICD shock treatment. Additionally, digitalis therapy positively altered the likelihood for rehospitalization due to cardiac reasons, leading to a significantly lower rehospitalization rate. The latter findings on the secondary endpoints were assessed in subgroups of the patients, and can therefore not be generalized to the whole patient cohort.

Nonetheless, our results suggest that, while digitalis may not influence overall mortality, its adverse impact on CRT efficacy and the heightened risk of arrhythmias necessitate caution in its use among CRT-D patients. Future research should focus on identifying specific patient subgroups that may benefit from digitalis without experiencing these negative effects. Moreover, advancements in monitoring and personalized medicine approaches could potentially mitigate the risks associated with digitalis therapy, optimizing its use in the context of modern heart failure management. Digitalis might be beneficial in a well-selected patient group, especially to reduce rehospitalization for cardiac reasons. Integrating genetic and biomarker analyses might also provide deeper insights into patient-specific responses to digitalis, paving the way for more tailored therapeutic strategies. Overall, these results underscore the need for a nuanced approach to digitalis use in heart failure patients receiving CRT-D therapy, balancing potential benefits against significant risks.

## Figures and Tables

**Table 1:** Patient characteristics at CRT-D implantation.

Variables	All Patients n = 552	Patients on Digitalis n = 219	Patients not on Digitalis n = 333	*p*-Value
**Age mean (SD) [years]**	67 (11)	64 (11)	69 (11)	<0.001
**Male gender n (%)**	429 (77)	170 (79)	257 (76)	n. s.
**Primary prevention n (%)** **Secondary prevention n (%)**	439 (79) 113 (21)	163 (76) 51 (24)	276 (82) 62 (18)	n. s.
**Ischemic heart disease n (%)** **Non-ischemic heart disease n (%)**	298 (54) 254 (46)	116 (54) 98 (46)	183 (54) 155 (46)	n. s.
**NYHA Classification n (%)** **0 + I** **II** **III** **IV**	17 (3) 133 (24) 349 (63) 53 (10)	4 (2) 38 (18) 153 (71) 18 (9)	13 (4) 95 (28) 194 (57) 35 (11)	0.002
**LVEF mean % (SD)**	25 (7)	23 (7)	27 (7)	<0.001
**Upgrade n (%)**	177 (32)	85 (40)	92 (27)	0.002
**Hypertension n (%)**	381 (69)	151 (71)	229 (68)	n. s.
**QRS width mean (SD) [ms]**	160 (28)	162 (31)	159 (27)	n. s.
**Atrial Fibrillation** **(including paroxysmal/persistent/permanent forms) n (%)**	200 (36)	101 (47)	98 (29)	<0.001
**Diabetes mellitus n (%)**	194 (35)	85 (40)	109 (32)	n. s.
**eGFR median (min–max) [ml/min]**	56 (6–138)	63 (20–138)	54 (6–120)	0.004
**Amiodarone n (%)**	117 (21)	52 (24)	65 (19)	n. s.

**Table 2:** (Heart failure) medication at CRT-D implantation.

Medication	All Patients n = 552	Patients on Digitalis n = 219	Patients Not on Digitalis n = 333	*p*-Value
**Beta-blocker n (%)**	533 (97)	207 (97)	326 (96)	n. s.
**ACE-Inhibitor/Angiotensin-Receptor Blocker n (%)**	524 (95)	204 (96)	320 (94)	n. s.
**Aldosterone antagonist n (%)**	397 (72)	152 (71)	254 (72)	n. s.
**Diuretics n (%)**	497 (90)	194 (91)	303 (89)	n. s.
**Statin n (%)**	368 (67)	142 (67)	226 (67)	n. s.
**Anticoagulation n (%)**	272 (49)	116 (55)	156 (46)	n. s.
**Antiplatelet Therapy n (%)**	309 (56)	105 (49)	204 (60)	0.01
**Amiodarone n (%)**	117 (21)	52 (24)	65 (19)	n. s.

**Table 3:** Independent predictors of mortality on multivariate Cox-regression analysis.

Parameter	Adjusted Hazard Ratio	95% Confidence Intervall	*p*-Value
**Age**	1.03	1.01–1.05	0.001
**Male Sex**	2.52	1.55–4.08	<0.001
**Chronic kidney disease**	1.72	1.22–2.42	0.002
**Higher NYHA classification**	1.47	1.15–1.89	0.002
**Upgrade**	1.36	1.00–1.89	0.07

**Table 4:** Cause of death classified according to Hinkle and Thaler model.

Death Cause	All n = 114	Patients on Digitalis n = 70	Patients Not on Digitalis n = 44	*p*-Value
**Cardiac non-arrhythmic n (%)**	39 (34)	24 (34)	15 (34)	0.57
**Cardiac arrhythmic n (%)**	21 (18)	13 (19)	8 (18)	0.53
**Other cardiovascular n (%)**	6 (5)	2 (3)	4 (9)	0.15
**Non-cardiovascular n (%)**	25 (22)	15 (21)	10 (23)	0.52
**Unknown n (%)**	23 (21)	16 (23)	7 (16)	0.28

## Data Availability

Data are contained within the article.

## Abbreviations

CRT	Cardiac resynchronization therapy
NYHA	New York Heart Association Classification for symptomatic heart failure
LVEF	Left ventricular ejection fraction
ICD	Implantable cardioverter-defibrillator
HR	Hazard ratio
CI	Confidence interval
HF	Heart failure
AF	Atrial fibrillation
SGLT-2	Sodium-glucose-uptake inhibitors
VT/VF	Ventricular tachycardia/ventricular fibrillation
ACE	Angiotensin-converting-enzyme
